# Data Flow Construction and Quality Evaluation of Electronic Source Data in Clinical Trials: Pilot Study Based on Hospital Electronic Medical Records in China

**DOI:** 10.2196/52934

**Published:** 2024-06-27

**Authors:** Yannan Yuan, Yun Mei, Shuhua Zhao, Shenglong Dai, Xiaohong Liu, Xiaojing Sun, Zhiying Fu, Liheng Zhou, Jie Ai, Liheng Ma, Min Jiang

**Affiliations:** 1 Key Laboratory of Carcinogenesis and Translational Research (Ministry of Education/Beijing), National Drug Clinical Trial Center, Peking University Cancer Hospital & Institute, Beijing, China; 2 Yidu Tech Inc, Beijing, China; 3 Pfizer (China) Research & Development Co, Shanghai, China; 4 State Key Laboratory of Holistic Integrative Management of Gastrointestinal Cancers, Beijing Key Laboratory of Carcinogenesis and Translational Research, National Drug Clinical Trial Center, Peking University Cancer Hospital & Institute, Beijing, China

**Keywords:** clinical trials, electronic source data, EHRs, electronic data capture systems, data quality, electronic health records

## Abstract

**Background:**

The traditional clinical trial data collection process requires a clinical research coordinator who is authorized by the investigators to read from the hospital’s electronic medical record. Using electronic source data opens a new path to extract patients’ data from electronic health records (EHRs) and transfer them directly to an electronic data capture (EDC) system; this method is often referred to as eSource. eSource technology in a clinical trial data flow can improve data quality without compromising timeliness. At the same time, improved data collection efficiency reduces clinical trial costs.

**Objective:**

This study aims to explore how to extract clinical trial–related data from hospital EHR systems, transform the data into a format required by the EDC system, and transfer it into sponsors’ environments, and to evaluate the transferred data sets to validate the availability, completeness, and accuracy of building an eSource dataflow.

**Methods:**

A prospective clinical trial study registered on the Drug Clinical Trial Registration and Information Disclosure Platform was selected, and the following data modules were extracted from the structured data of 4 case report forms: demographics, vital signs, local laboratory data, and concomitant medications. The extracted data was mapped and transformed, deidentified, and transferred to the sponsor’s environment. Data validation was performed based on availability, completeness, and accuracy.

**Results:**

In a secure and controlled data environment, clinical trial data was successfully transferred from a hospital EHR to the sponsor’s environment with 100% transcriptional accuracy, but the availability and completeness of the data could be improved.

**Conclusions:**

Data availability was low due to some required fields in the EDC system not being available directly in the EHR. Some data is also still in an unstructured or paper-based format. The top-level design of the eSource technology and the construction of hospital electronic data standards should help lay a foundation for a full electronic data flow from EHRs to EDC systems in the future.

## Introduction

Source data are the original records from clinical trials or all information recorded on certified copies, including clinical findings, observations, and records of other relevant activities necessary for the reconstruction and evaluation of the trial [1]. Electronic source data are data initially recorded in an electronic format (electronic source data or eSource) [2,3].

The traditional clinical trial data collection process requires a clinical research coordinator (CRC) who is authorized by the investigators to read from the hospital’s electronic medical record and other clinical trial–related data from the hospital information system and then manually enter the patient’s data into the electronic data capture (EDC) system. After data entry, the clinical research associate visits the site to perform source data verification and source data review. The drawbacks of collecting data by manual transcription are that data quality and timeliness cannot be guaranteed and that it is a waste of human and material resources. Using electronic source data opens a new path to extract patients’ data from electronic health records (EHRs) and transfer it directly to EDC systems (often the method is referred to as eSource) [4]. eSource technology in a clinical trial data flow can improve data quality without compromising timeliness [5]. At the same time, improved data collection efficiency reduces clinical trial costs [6].

eSource can be divided into two levels. The first level is to enable the hospital information system to obtain complete data sets; the second level is to allow direct data transfer to EDC systems based on the clinical trial patients’ electronic data in hospitals to avoid the electronic data being transcribed manually again, which is the core purpose of eSource [7]. This project will explore the use of eSource technology to extract clinical trial data from EHRs, send it to the sponsor data environment, and discuss the issues and challenges occurring in its application process.

## Methods

### Ethics Approval

This study was approved by the Ethics Committee and Human Genetic Resource Administration of China (2020YW135). During the ethical review process, the most significant challenges were patients’ informed consent, privacy protection, and data security. The B7461024 Informed Consent Form (Version 4) states that *“*interested parties may use subjects’ personal information to improve the quality, design, and safety of this and other studies,” and “Is my personal information likely to be used in other studies? Your coded information may be used to advance scientific research and public health in other projects conducted in future.” This project is an exploration of using electronic source data technology instead of traditional manual transcription in the process of transferring data from hospital EHRs to EDC systems, which will improve the data quality of clinical trials and will improve the data flow in the future. Therefore, this project is within the scope of the informed consent form for study B7461024, which was approved by the ethics committee after clarification.

### Project Information

This project was conducted from December 15, 2020, to November 19, 2021, which was before China’s personal information protection law and data security law were introduced. The data for this project were obtained from an ongoing phase 2, multicenter, open-label, dual-cohort study to evaluate the efficacy and safety of Lorlatinib (pf-06463922) monotherapy in anaplastic lymphoma kinase (ALK) inhibitor–treated locally advanced or metastatic ALK-positive non–small cell lung cancer patients in China (B7461024), registered by the sponsor on the Drug Clinical Trials Registration and Disclosure Platform (CTR20181867). The data extraction involved 4 case report form (CRF) data modules: demographics, concomitant medication, local lab, and vital signs, which were collected in the following ways:

- Demographics: Originally entered directly into the hospital EHR then manually transcribed by the CRC to the sponsor’s EDC system
- Local lab: Laboratory data collected by the hospital laboratory information management system (LIMS) and then manually transcribed by the CRC into the EDC system
- Vital signs: Hospital uses paper-based tracking form provided by the sponsor to record patients’ vital signs and investigators transcribe the vital signs data into the hospital medical record
- Concomitant medication: Similar to vital signs, hospital uses the paper tracking form provided by the sponsor to record the adverse reactions and concomitant medication; investigator might also transfer the concomitant medication data into the hospital EHR, but there was no mandatory requirement to transfer these data into patients’ medical records

All information was collected from 6 patients in a total of 29 fields (Textbox 1).

Textbox 1. **Data collection fields.**

**Demographics**

- Subject ID
- Date of birth
- Sex
- Ethnicity
- Race
- Age

**Concomitant medication**

- Combined drug name
- Whether for the treatment of adverse reactions
- Adverse event number
- Combined drug start date
- Combined drug end date
- Currently still in use

**Vital signs**

- Date of vital signs collection
- Weight
- Weight unit
- Body temperature
- Height
- Height unit
- Location of temperature measurement
- Systolic blood pressure
- Diastolic blood pressure
- Pulse

**Local lab**

- Laboratory inspection name
- Laboratory name and address
- Sponsor number
- Laboratory number
- Incomplete laboratory inspection
- Sample collection data
- Inspection results

### Data Process Workflow

#### Overview

The study chosen in our project used the traditional manual data entry method to transcribe patients’ CRF data into the EDC system. This project proposes testing the acquisition of data directly from the hospital EHR, deidentification of the patients’ electronic data on the hospital medical data intelligence platform, mapping and transforming the data based on the sponsor’s EDC data standard, and transferring the data into the sponsor’s environment. The data was transferred from the hospital to the sponsor’s data environment and compared to data that was captured by traditional manual entry methods to verify the availability, completeness, and accuracy of the eSource technology.

In the network environment of this project, the technology provider accessed the hospital network through a virtual private network (VPN) and a bastion host, and processed the data of this project as a private cloud, thus ensuring the security of the hospital data.

#### Data Integration

The hospital information system involved in this project has reached the national standards of “Level 3 Equivalence,” “Electronic Medical Record Level 5,” and “Interoperability Level 4.” The medical data intelligence platform in this project is deployed in a hospital intranet, isolated from external networks. Integrated data from different information systems, including the hospital information system, LIMS, picture archiving and communication system, etc, were deidentified from the platform and transferred to a third-party private cloud platform for translation and data format conversion after authorization by the hospital through a VPN.

The scope of data collection in this project was limited to patients who signed Informed Consent Form (Version 4) for study B7461024. The structured data of four CRF data modules (demographic, concomitant medications, local lab, and vital signs) were extracted from the source data in hospital systems, and data processing was completed.

#### Three-Layer Deidentification of Data

In this project, three layers of deidentification were performed on the electronic source data to ensure data security. The first layer of deidentification was performed before the certified copy of data was loaded to the hospital’s medical data intelligence platform. The second layer of deidentification follows the Health Insurance Portability and Accountability Act (HIPAA) by deidentifying 18 data fields at the system level. A third layer of deidentification was performed when mapping and transforming third-party databases for the clinical trial data (demographics, concomitant medications, laboratory tests, and vital signs) collected for this study, as required by the project design.

Collected data did not contain any sensitive information with personal identifiers of the patients, and all deidentification processes were conducted in the internal environment of the hospital. In addition to complying with the relevant laws and regulations, we followed the requirements of Good Clinical Practice regarding patient privacy and confidentiality, and further complied with the requirements of HIPAA to deidentify the 18 basic data fields. Data fields outside the scope of HIPAA will be deidentified and processed in accordance with the TransCelerate guidelines published in April 2015 to ensure the security of patients’ personal information and to eliminate the possibility of patient information leakage [8].

The general rules for the third layer of deidentification were as follows:

- Time field: A specific time point is used as the base time, and the encrypted time value is the difference between the word time and the base time
- ID field: Categorized according to the value and only shows the category
- Age field: Categorized according to the value and only shows the category
- Low-frequency field: set to null

In addition, all data flows keep audit trails throughout and are available for audit.

#### Data Normalization and Information Extraction

After three layers of deidentification, the data was transferred from a hospital to a third-party private cloud platform through a VPN, where translation from Chinese to English and data format conversion were implemented. The whole transfer process was performed for the data that was collected for the clinical trial of this study. Standardization of data is a crucial task during the data preparation phase. This process involves consolidating data from different systems and structures into a consistent, comprehensible, and operable format. First, a thorough examination of data from various systems is necessary. Understanding the data structure, format, and meaning of each system is essential. The second step involves establishing a data dictionary that clearly outlines the meaning, format, and possible values of each data element. Next, selecting a data standard is necessary to ensure consistency and comparability. In this study, we adopted the Health Level 7 (HL7) standard. Additionally, data cleansing and transformation are needed to meet standard requirements, including handling missing data, resolving mismatched data formats, or performing data type conversions. Extract, transform, and load tools were used to integrate data from different systems. Data security must be ensured throughout the data integration process. This includes encrypting sensitive information and strictly managing data permissions. Data verification and validation steps were then performed by professional staff on the translated data. The data from the hospital’s medical data intelligence platform were then converted from JSON format to XML and Excel formats. The processed data was transferred back to the hospital via a VPN to a designated location for final adjudication before loading to the sponsor’s environment.

#### One-Time Data Push and Quality Assessment

**Figure 1.**
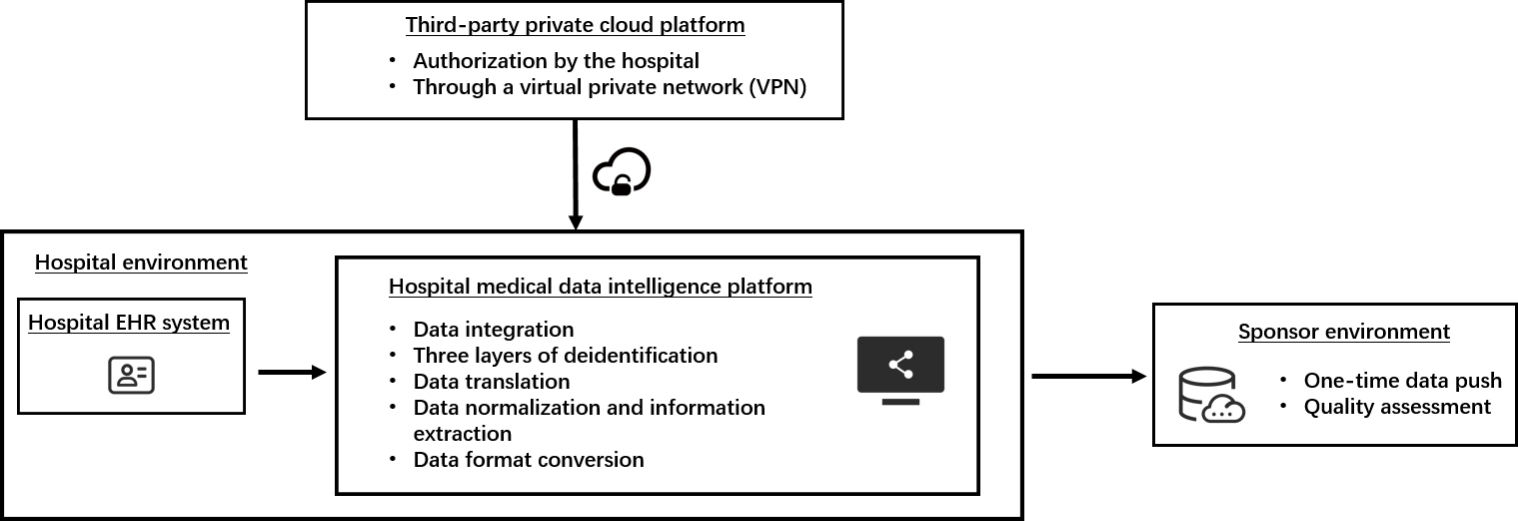
Project operation flow. EHR: electronic health record.

After the hospital received the processed data, it was then pushed by the hospital to the sponsor’s secure and controlled environment (Figure 1). All data deidentification processes were conducted in the hospital’s environment, and none of the data obtained by the sponsor can be traced back to patients’ personal information to ensure their privacy and information security.

The data quality of this project was assessed using industry data quality assessment rules [9], which are shown in Table 1.

**Table 1.** Introduction of data quality assessment rules.

Data validation methods	Dimension	Method description	Cases
Data availability verification	Field dimension	The ratio of the total number of data fields in the clinical trial CRF^a^ available in the hospital EHR^b^ to the total number of data fields required in the electronic CRF: EHR^c^/CRF^d^ × 100%	Based on the electronic CRF, 6 data fields in the demography need to be captured, and 3 of them have records in the EHR. Data availability: 3/6 × 100% = 50%
Data availability verification	Field dimension	The ratio of the total number of data fields in the clinical trial CRF (eSource) that can be transmitted electronically in the hospital’s EHR to the total number of data fields required in the electronic CRF: eSource^e^/CRF^d^ × 100%	Based on the electronic CRF, 6 data fields in the demography need to be captured, and 2 data fields can be captured by the eSource method. Data availability: 2/6 × 100% = 33.33%
Data completeness verification	Numerical dimension	The ratio of the total number of nonnull data (eSourceV) captured (processed and sent to the sponsor) via the eSource method to the total number of data fields requested on the electronic CRF: eSourceV^f^/CRF^d^ × 100%	Based on the clinical trial design, 38 concomitant medication pages need to be collected: 7 pages were collected via eSource and 2 fields was entered per page. Data completeness: 7 × 2/(2 × 38) × 100% = 18.42%
Data accuracy verification	Numerical dimension	Matching of data field values in the hospital’s EHR with data field values that can be captured by eSource (data fields that are processed and sent to the sponsor)	4 fields of demography were successfully transmitted through eSource, with 4 data points in each. After comparing with the data in the electronic data capture system, there were no mismatches for one data point. Data accuracy: 8/(2 × 4) × 100% = 100%

a CRF: case report form.

b EHR: electronic health record.

c Total number of data fields in the hospital’s EHR.

d Total number of data fields requested in the electronic CRF.

e Total number of data fields captured (processed and sent to the sponsor) through the eSource method.

f Total number of nonempty data fields captured (processed and sent to the sponsor) through the eSource method.

## Results

In this project, we collected patients’ demographics, vital signs information, local laboratory data, and concomitant medication data from EHRs, successfully pushed the data directly to the designated sponsor environment, and evaluated the data quality from three perspectives including availability, completeness, and accuracy (Table 2).

- The eSource-CRF availability score, which is used to evaluate the ratio of fields in EHR that can be collected by eSource and used for CRF, was low for demographics, blood tests, and urine sample tests but higher for vital signs and concomitant medications.
- Data completeness, defined as the ratio of the total number of nonnull data captured by eSource to the total number of data fields required in the electronic CRF, was used to evaluate the ratio of nonnull data fields in the CRF that can be captured by eSource. In this study, the completeness score of the vital signs module was only 1.32%, and the concomitant medications and laboratory test modules also had poor performance in the data completeness evaluation.
- Data accuracy, defined as the compatibility between the data field values in the hospital EHR and the data field values that can be collected using eSource, was 100% for all modules.

- EHR-CRF availability, which is used to evaluate the ratio of fields in the EHR that can be used for the CRF, was 50%, 60%, and 66.67% for demographics, blood tests, and urine sample tests, respectively, in this study, and the rest of the data were 100% available.

**Table 2.** Metrics measured.

CRF^a^ domain		CRF-EHR^b^ data availability, n/N (%)	CRF-eSource data availability, n/N (%)	Data completeness (preliminary findings), n/N (%)	Data accuracy (preliminary findings), n/N (%)
Definition		Study CRF data elements available in hospital EHR	Study CRF data elements available in hospital EHR and able to be electronically transferred through eSource technology	Study CRF data elements available and entered into hospital EHR and transferred through eSource technology	Study CRF data elements available and entered into hospital EHR and transferred through eSource technology with expected result (eg, matches what was entered directly in form)
Demographics		3/6 (50.00)	2/6 (33.33)	12/12 (100.00)	12/12 (100.00)
Vital signs		10/10 (100.00)	9/10 (90.00)	24/1812 (1.32)^c^	20/20 (100.00)
**Local lab**					
	Blood biochemical tests	6/10 (60.00)	5/10 (50.00)	12,968/13,540 (95.78)^d^	7767/7767 (100.00)
	Urine sample tests	6/9 (66.67)	5/9 (55.56)	15/40 (37.56)	15/15 (100.00)
Concomitant medication		10/10 (100.00)	9/10 (90.00)	14/76 (18.42)^e^	6/6 (100.00)

a CRF: case report form.

b EHR: electronic health record.

c Checks were made with the relevant clinical research associates (CRAs) regarding the original data collection and CRF completion methods for the following reasons: vital signs were obtained using paper tracking forms provided by the sponsor as the original data source, and the data may not be transcribed into the hospital information system (HIS) by the researcher. Therefore, data from many visits are not available in the HIS.

d A total of 2708 blood biochemistry tests were involved.

e Concomitant medication uses tracking forms to record adverse event and ConMed (a paper source), and data may not be transcribed into the HIS. As confirmed by the CRA, the percentage of paper ConMed sources was approximately 80%.

## Discussion

Although EHRs have been widely used, the degree of structure of EHR data varies substantially among different data modules. In EHRs, demographics, vital signs, local lab data, and concomitant medications are more structured than patient history or progress notes and often contain unstructured text [10]. Therefore, we selected these 4 well-structured data modules for exploration in this project.

For demographics data, among the 6 required fields (subject ID, date of birth, sex, ethnicity, race, and age), subject ID (subject code number/identifier in the trial, not the patient code number/identifier in the EHR system), ethnicity, and race were not available in the EHR, so the EHR-CRF availability score was 50%. Since this was an exploratory project, the date of birth field was also deidentified and thus could not be collected based on our deidentification rule, so the eSource-CRF availability score was 33%. In the future, the availability score can reach close to 100% by bidirectional design of the EHR and CRF under the premise of obtaining compliance for industrial-level applications.

The low availability score of local laboratory data on EHR-CRFs is due to the lack of required fields in the hospital system; “Lab ID” and “Not Done” do not exist in the LIMS, and for the “Clinically Significant” field, the meaning of laboratory test results needs to be manually interpreted by an investigator, so they cannot be transcribed directly. The availability score of eSource-CRFs was further decreased because the field “Laboratory Name and Address” is not an independent structured field in the EHR. The completeness score of urine sample test data was only 37.56% because during the actual clinical trial, especially amid the COVID-19 pandemic period, patients completed study-related laboratory tests at other sites, and those test results were collected via paper-based reports, so the complete data sets cannot be extracted from the site’s system.

To improve data availability in future applications, clinical trial–specific fields need to be added to EHR designs for those data that require an investigator’s interpretation such as “Clinically Significant,” and data transfer and mapping processes for the determination of the scope of data collection also needs to be optimized. Based on these two conditions, the completeness score can be improved to over 90%.

The availability and accuracy of vital signs data are ideal. However, since not all vital signs data collection was recorded by the electronic system during the actual study visit, many vital signs data were collected in “patient diary” and other types of paper-based documents during the study, resulting in a serious limitation in data completeness. With the development of more clinical trial–related electronic hardware and enhancements in products intelligence, more vital signs data will be directly collected by electronic systems, and the completeness of vital signs data transferred from EHR to EDC will be greatly improved in the future.

In the concomitant medication module, there was a good score for availability and accuracy because the standardization and structuring of prescriptions are well done in this hospital system. However, the patient’s medication use period during hospitalization is recorded in unstructured text, so the data could not be captured for this study, resulting in a low completeness score of 18.42% for concomitant medication.

In summary, the accuracy score of eSource data in this study was high (100% for all fields). A study by Memorial Sloan Kettering Cancer Center and Yale University confirmed that the error rate of automatic transcription reduced from 6.7% to 0% compared to manual transcription [10]. However, data availability and completeness have not reached a good level. Data availability varies widely across studies, ranging from 13.4% in the Retrieving EHR Useful Data for Secondary Exploitation (REUSE) project [11] to 75% in The STARBRITE Proof-of-Concept Study [12], mainly related to the coverage and structure of the EHR.

National drug regulatory agencies (eg, US Food and Drug Administration [FDA], European Medicines Agency, Medicines and Healthcare products Regulatory Agency, and Pharmaceuticals and Medical Devices Agency) have developed guidelines to support the application of eSource to clinical trials [3,13-15]. The new Good Clinical Practice issued by the Center for Drug Evaluation in 2020 encourages investigators to use clinical trials’ electronic medical records for source data documentation [1]. Despite this, we still encountered challenges, including ethical review and data security, during this study’s implementation process. Without knowing the precedents, the project team decided to follow the requirements for clinical trials to control the quality of the study. There were no existing regulatory policies or national guidance on eSource in China at the time of this study. The project team provided explanations for inapplicable documents and communicated several times to ensure the approval of relevant institutional departments before finally becoming the first eSource technology study to be approved by the Ethics Committee and Human Genetic Resource Administration of China.

In the absence of regulatory guidelines, our eSource study, the first in China’s International Multi-center Clinical Trial, navigated challenges in data deidentification. We adopted HIPAA and TransCelerate’s guidelines [8]. Securing approval under “China International Cooperative Scientific Research Approval for Human Genetic Resources,” we answered queries and achieved unprecedented recognition. For transferring data from the hospital to the sponsor’s environment, we prioritized security and obtained necessary approvals. Iterative revisions ensured a robust data flow design. Challenges in mapping hospital EHR to EDC standards highlighted the need for a scalable mechanism. This study pioneers eSource tech integration in China, emphasizing the importance of seamless data mapping. In the process of executing data standardization, several challenges may arise, including inconsistent data definitions. Data from different systems may use different definitions due to the independent development of these systems, leading to varied interpretations of even identical concepts. To address this issue, establishing a unified data dictionary is crucial to ensure consensus on the definition of each data element. Different systems might also use distinct data formats such as text encodings. Preintegration format conversion is required, and extract, transform, and load tools or scripts can assist in standardizing these formats. During the integration of data from multiple systems, it is possible to discover data in one system that is not present in another. In the data standardization process, considerations must be made on how to handle missing data, which may involve interpolation, setting default values, etc. Quality issues like errors, duplicates, or inaccuracies may exist in data from different systems. Data cleansing, involving deduplication, error correction, logical validation, etc, is necessary to address these quality issues. Different systems may generate data based on diverse business rules and hospital use scenarios. In data standardization, unifying these rules requires collaboration with domain experts to ensure consistency.

Internationally, multiple research studies and publications have been released on regulations, guidelines, and validation of eSource. The FDA provided guidance on the use of electronic source data in clinical trials in 2013 that aims to address barriers to capturing electronic source data for clinical trials, including the lack of interoperability between EHRs and EDC systems. The European-wide Electronic Health Records for Clinical Research (EHR4CR) project was launched in 2011 to explore technical options for the direct capture of EHR data within 35 institutions, and the project was completed in 2016 [16]. The second phase of the project connected the EHRs to EDC systems [17] and aimed to realize the interoperability of EHRs and EDC systems. The US experience focuses more on improving and standardizing the existing EHRs to make them more uniform.

In Europe, the experience focuses on breaking down the technical barrier of interoperability between EHRs and EDC systems. In China, the current industry trends focus on the governance of existing EHR data in the hospital and the building of clinical data repository platforms [7]. Clinical data repository platforms focus on data integration and cleaning between EHRs and other systems in hospital environments and on unstructured data normalization and standardization by natural language processing and other AI technology [18]. At the national level, China is also actively promoting the digitization of medical big data and is committed to the formation of regional health care databases [19], which lays the foundation for the future implementation of eSource in China [20].

This study evaluates the practical application value of eSource in terms of availability, completeness, and accuracy. To improve availability, the structure of the CRF needs to be designed according to the information of the EHR data at the design stage of clinical trials. Even so, since EHRs are designed for the physicians to conduct daily health care activities, certain fields in clinical trials (eg, judgment of normal or abnormal values of laboratory tests and judgment of correlations of adverse events and combined medications) are still not available, and clinical trial–specific fields need to be added to EHR designs for those data that require investigators’ interpretation to improve data availability. Completeness could be improved by the development of hospital digitalization that ensures patients’ data is collected electronically rather than on paper. Additionally, 2708 blood test records were successfully collected from only 6 patients via eSource in this study, which indicates that laboratory tests often contain large amounts of highly structured data that are suitable for eSource. EHR-EDC end-to-end automatic data extraction by eSource is suitable for laboratory examinations and can improve the efficiency and accuracy of data extraction significantly as well as reduce redundant manual transcriptions and labor costs. Processing unstructured or even paper-based data in eSource is still a big challenge. Using machine learning tools (eg, natural language processing tools) for autostructuring can be explored in the future. The goal is to have common data standards and better top-level design to facilitate data integrity, interoperability, data security, and patient privacy protection in eSource applications. During deidentification, we processed certain data with a specific logic to protect privacy. The accuracy assessment was performed during the deidentification step to ensure that the data was still sufficiently accurate while meeting privacy requirements. Reversible methods need to be used when performing deidentification as well as providing controlled access mechanisms to the data so that the raw data can be accessed when needed. It is worth noting that different regions and industries may have different privacy regulations and compliance requirements. When deidentifying, you need to ensure that you are compliant with the relevant regulations and understand the limitations of data use. This may require working closely with a legal team.

In the future, we can consider adding performance analysis, including an assessment of data import performance. This involves evaluating the speed and efficiency of data import to ensure it is completed within a reasonable timeframe. Additionally, analyzing data query performance is crucial in practical applications to ensure that the imported data meets the expected query performance in the application. For long-term applications involving a larger size of patients, it is advisable to consider adding analyses related to maintainability and cost-effectiveness. This includes implementing detailed logging and monitoring mechanisms to promptly identify and address potential issues. Furthermore, for the imported data, establishing a version control mechanism is essential for tracing and tracking changes in the data. Simultaneously, for overall resource use, evaluating the resources required during the data import process ensures completion within a cost-effective framework. It is also important to consider the value of imported data for clinical trial operations and related decision-making, providing a comparative analysis between cost and value.

## Abbreviations

ALK: anaplastic lymphoma kinase
CRC: clinical research coordinator
CRF: case report form
EDC: electronic data capture
EHR: electronic health record
EHR4CR: Electronic Health Records for Clinical Research
FDA: Food and Drug Administration
HIPAA: Health Insurance Portability and Accountability Act
HL7: Health Level 7
LIMS: laboratory information management system
REUSE: Retrieving EHR Useful Data for Secondary Exploitation
VPN: virtual private network
